# Ferulic acid inhibits lipogenesis and ameliorates MASLD via targeting PGC-1β

**DOI:** 10.3389/fnut.2025.1730916

**Published:** 2026-01-05

**Authors:** Kaili Cui, Laifeng Ren, Lichao Zhang, Yuxuan An, Pengyu Ji, Ye Yang, Feng Li, Zhuoyu Li

**Affiliations:** 1 Central Laboratory, Shanxi Province Cancer Hospital/Shanxi Hospital Affiliated to Cancer Hospital, Chinese Academy of Medical Sciences/Cancer Hospital Affiliated to Shanxi Medical University, Taiyuan, China; 2 The Key Laboratory of Chemical Biology and Molecular Engineering of Ministry of Education, Institute of Biotechnology, Shanxi University, Taiyuan, China; 3 Institutes of Biomedical Sciences, Shanxi University, Taiyuan, China

**Keywords:** ferulic acid, MASLD, PGC-1β, SREBP1, lipogenesis

## Abstract

**Introduction:**

Metabolic dysfunction-associated steatotic liver disease (MASLD) is characterized by hepatic steatosis and increased triglyceride content. Thus, intervention in fatty acid metabolism is very desirable for NAFLD treatment. Ferulic acid (FA) is a plant-derived bioactive molecule that regulates lipid metabolism.

**Methods:**

High-fat diet (HFD)-fed mice and free fatty acid (FFA) -treated cells were used to evaluate the improvement of FA. The target proteins of FA were screened by solid phase extraction combined with mass spectrometry.

**Results:**

It was found that FA effectively improved MASLD in vivo and in vitro. Interestingly, PPAR gamma-coactivator-1beta (Ppargc1β, also known as PGC-1β) was the target of FA intervention in MASLD. FA directly bound to PGC-1β and inhibited its expression through the ubiquitin-proteasome pathway. Furthermore, Overexpression of PGC-1β abolished the ameliorative effect of FA on MASLD. In addition, FA inhibited lipogenesis through the PGC-1β/SREBP1 axis, thereby improving MASLD.

**Discussion:**

This work uncovered a novel plant-derived therapeutic strategy targeting a previously unrecognized PGC-1β/SREBP1 mechanism in MASLD.

## Graphical abstract

**Figure:**
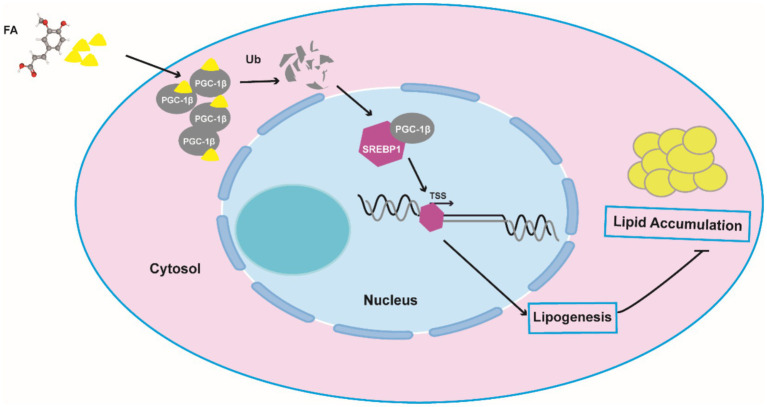
Diagram illustrating the molecular mechanism of FA ameliorates MASLD. FA directly binds to and inhibits the expression of PGC-1β protein through the ubiquitin–proteasome pathway. FA inhibits lipogenesis through the PGC-1β/SREBP1 axis, thereby improving MASLD. Hence, PGC-1β serves as an underlying target for FA to improve MASLD.

## 1 Introduction

Metabolic dysfunction-associated steatotic liver disease (MASLD) is a leading cause of liver dysfunction and is rapidly becoming the most prevalent chronic liver disease worldwide (1). Its prevalence in the general population ranges between 20 and 30%, and the incidence is still rising (2). The disease spectrum of MASLD, ranging from simple steatosis to MASH, further develops fibrosis and progresses to cirrhosis and hepatocellular carcinoma. Although MASLD has received increasing attention due to its high prevalence and progressive pathological features, few effective pharmacotherapies have been approved (3). To date, the U.S. Food and Drug Administration (FDA) has approved only two drugs specifically for MASH: resmetirom (approved in 2024) and semaglutide (approved in 2025), both under accelerated approval pathways and intended to be used in conjunction with dietary control and increased physical activity.

MASLD is characterized by lipid droplet accumulation and elevated triglyceride (TG) levels in the liver. As the central regulator of lipid homeostasis, the liver is responsible for coordinating the balance between lipid acquisition and lipid disposal (4). Dyslipidemia leads to excessive deposition of free fatty acids (FFAs) into triglycerides in the liver, contributing to hepatic steatosis and the subsequent progression of MASLD (5). Therefore, reducing lipid accumulation is considered to be the key therapeutic strategy for MASLD. Among the key regulators of hepatic lipogenesis, the transcriptional coactivator PGC-1β has emerged as a critical player. It has been reported that PGC-1β acts as a transcriptional activator and activates transcription factors in a ligand-dependent manner to play a role in transcriptional regulation (6). It is also known to physically interact with and coactivate sterol regulatory element-binding protein 1 (SREBP1), a master regulator of genes involved in triglyceride synthesis (7). This PGC-1β/SREBP1 axis represents a central pathway driving *de novo* lipogenesis.

Ferulic acid (the chemical structure is shown in Supplementary Figure S1) is a phenolic acid compound commonly found in food and various traditional Chinese medicines. It is naturally present in wheat, brown rice, ferula, Ligusticum chuanxiong, and *Angelica sinensis* (8). FA has a wide range of pharmacological activities, including anti-obesity (9), antioxidant (10), and anti-inflammatory (11) effects, which have been used to prevent metabolic diseases (12, 13). In recent years, FA has been reported to improve blood pressure in diet-induced hypertensive rats by regulating the hepatic lipid metabolic profile (14). The protective effect of ferulic acid was also observed in diabetic rats through the alleviation of lipid peroxidation (15). These studies have demonstrated that FA can ameliorate key pathological features of the disease, including reducing hepatic steatosis, improving insulin sensitivity, and mitigating oxidative stress and inflammation in various experimental models. These findings highlight FA as a promising candidate for NAFLD intervention. Building upon this foundation, the present study aimed to further elucidate the underlying mechanisms through which FA exerts its protective effects. Specifically, we focused on the target and underlying molecular mechanism of FA in MASLD. The study found that FA inhibited its target protein PGC-1β, thereby regulating lipogenesis and improving MASLD. Our research provides new insights into the molecular mechanisms of FA in NAFLD and offers experimental evidence supporting its potential as a therapeutic agent.

## 2 Materials and methods

### 2.1 Materials

Ferulic acid (FA) was purchased from Victory Biological Technology Co., Ltd. (Sichuan, China). Dulbecco’s Modified Eagle Medium (DMEM) and fetal bovine serum (FBS) were purchased from Biological Industries (Kibbutz Beit Haemek, Israel). Oil Red O staining test kits, 4,4-difluoro-4-bora-3a,4a-diaza-s-indacene (BODIPY), 4′,6-diamidino-2-phenylindole (DAPI), 3-(4,5-dimethylthiazol-2-yl)-2,5-diphenyltetrazolium bromide (MTT), and dimethyl sulfoxide (DMSO) were purchased from Solarbio (Beijing, China). Assay kits for total TG, cholesterol (TC), alanine aminotransferase (ALT), and aspartate aminotransferase (AST) were purchased from Nanjing Jiancheng Bioengineering Institute (Nanjing, China). Antibodies for PGC-1β and glyceraldehyde-3-phosphate dehydrogenase (GAPDH) were purchased from ProteinTech (Chicago, IL, USA). RNAiso Plus was purchased from Takara (Shiga, Japan). The TransScript First-Strand cDNA Synthesis SuperMix and TransStart Top Green quantitative reverse transcription polymerase chain reaction (qRT-PCR) SuperMix were purchased from TransGen (Beijing, China). TurboFect was purchased from Thermo Scientific (Waltham, MA, USA).

### 2.2 Mice experiment

This animal experiment was approved by the Ethics Committee of Animal Experimentation of Shanxi University (SXULL2020046). A total of 20 male C57BL/6J mice (5 weeks old), weighing 20 ± 5 g, were purchased from Beijing Vital River Laboratory Animal Technology Co., Ltd. The mice were housed in a specific pathogen-free room at 22 ± 2 °C under a 12:12-h light–dark cycle.

The mice were randomly divided into four groups: one control group and three treatment groups, with five mice per group. The control group was fed a normal diet, while the three treatment groups were fed a high-fat diet (HFD). Among the HFD groups, one was given an equal volume of water, one was given simvastatin (5 mg kg^−1^ day^−1^), and one was fed FA (30 mg kg^−1^ day^−1^). At the end of the study, all mice were anesthetized with ether, and their tissues were immediately collected, weighed, and frozen in liquid nitrogen for further analysis.

### 2.3 Cell culture and steatosis induction

The PLCPRF5, Hepatocellular Carcinoma, Grade 2 (HepG2), and Beijing Institute of Liver Cancer (BEL-7402) BEL-7402 cell lines were obtained from the Chinese Type Culture Collection (Shanghai, China). The cells were cultured in DMEM supplemented with 10% FBS and antibiotics at 37 °C in a humidified atmosphere containing 5% CO_2_. FFA (a mixture of oleic acid and palmitic acid at a ratio of 2:1) was used to treat hepatocytes to establish the MASLD model. For steatosis induction, HepG2 and PLCPRF5 cells were treated with 1 mM FFA for 24 h, whereas BEL-7402 cells were treated with 0.5 mM FFA for 24 h.

### 2.4 Solid-phase extraction assay

The solid-phase extraction assay was performed as described previously (16). PLCPRF5 or HepG2 cell lysates were incubated with FA crystallization at 4 °C overnight, and the supernatant was removed by centrifugation. The precipitated complex was washed with PBS to remove unbound proteins. The interaction between the PGC-1β protein and FA was then assessed.

### 2.5 Molecular docking

The crystal structure of PGC-1β (ID: AF-Q86YN6-F1) was obtained from the protein database and downloaded in Protein Data Bank (PDB) format. The small-molecule structure of FA (Compound CID: 445858) was downloaded from the PubChem database. AutoDock Vina (17) was used to simulate the binding of FA with PGC-1β.

### 2.6 Drug affinity responsive target stability (DARTS) assay

DARTS assays were performed according to a previously described protocol (18). PLCPRF5 cells were incubated in 10-cm dishes to 80% confluence and lysed in RIPA buffer containing a protease inhibitor. After centrifugation, the sediment was collected. The bicinchoninic acid (BCA) method was used to detect protein concentration. Each sample was divided into two aliquots, one for proteolysis with pronase and the other for mock proteolysis. Western blot analysis was performed after digestion. The concentration of FA was 40 μg/mL.

### 2.7 Cellular thermal shift assay (CETSA)

CETSA was performed according to a previously described protocol (19). The concentration of FA was 40 μg/mL.

### 2.8 Cycloheximide (CHX) chase assay

To observe the protein degradation process, PLCPRF5 and HepG2 cells were treated with cycloheximide (CHX) (20 μg/mL). Total protein was extracted at the specified time points and analyzed using Western blotting.

### 2.9 *In vitro* ubiquitination assay

The cells were treated with MG132 (20 μM) for 6 h before collection. Cell lysates were then prepared, immunoprecipitated using a PGC-1β antibody, eluted with 20 μL of lysis buffer, and denatured with SDS sample buffer. Ubiquitination modification of PGC-1β was assessed using Western blotting.

### 2.10 Quantitative RT-PCR assay

Quantitative reverse transcription-polymerase chain reaction (RT-PCR) assay was used to measure mRNA levels. Total RNA was extracted from cells and tissues using Trizol reagent. RNA was reverse-transcribed using an all-in-one first-strand cDNA synthesis kit. mRNA levels were quantified by qRT-PCR. GAPDH mRNA was used as the control. Relative expression was calculated using the 2^−ΔΔCt^ method. Primer sequences are listed in Supplementary Table S1.

### 2.11 Statistical analysis

The data are presented as the mean ± SEM. Analysis of variance (ANOVA) with *post-hoc* Tukey’s testing was used to compare the means of ≥3 groups. Univariate comparison was performed using Student’s *t*-test. *p*-values of <0.05 were considered statistically significant.

## 3 Results

### 3.1 FA attenuates MASLD in HFD-induced mice

**Figure 1:**
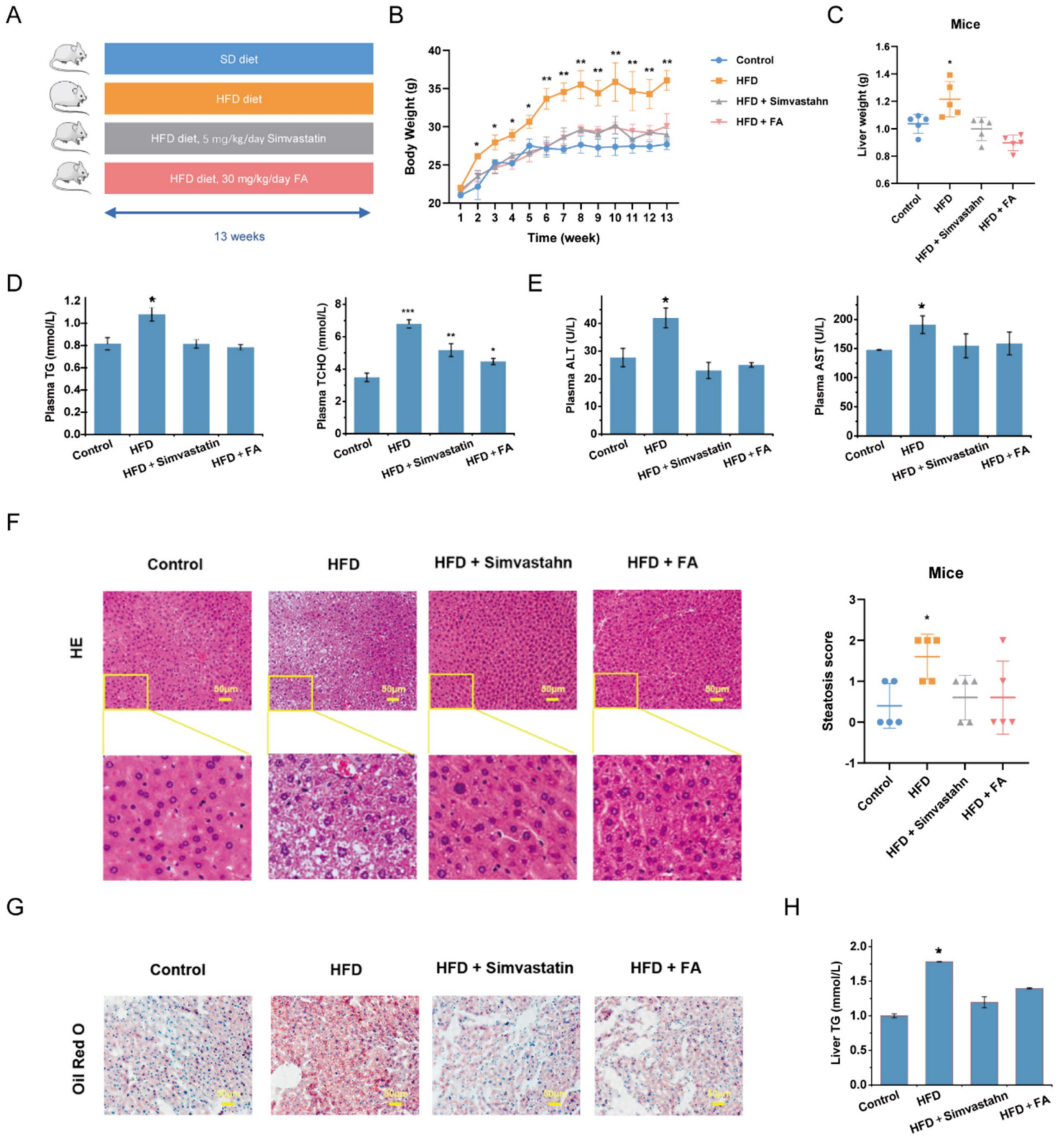
Effects of FA on HFD-induced hepatic steatosis. **(A)** An overview of the experimental design of the HFD-induced MASLD mouse model (*n* = 5). **(B)** Body mass. **(C)** Liver weight. **(D)** Serum total TG and TC. **(E)** Serum ALT and AST. **(F)** Representative images of H&E staining (scale bar = 50 μm) and MASLD activity score. **(G)** Representative images of Oil Red O staining (scale bar = 50 μm). **(H)** Liver TG. The data are presented as the mean ± SEM. *p < 0.05, **p < 0.01, ***p < 0.001.

To determine the effects of FA on the regulation of MASLD, an HFD-induced mouse model was used. It was found that FA significantly reduced body weight and liver weights compared to HFD diet-fed mice (Figures 1A–C; Supplementary Figure S2A). Food intake was not altered by FA (Supplementary Figure S2B). Meanwhile, the FA supplementation reduced the levels of serum total TG and TC, as well as the levels of serum ALT and AST (Figures 1D,E). Histologically, hematoxylin and eosin (H&E) and Oil Red O staining revealed the presence of hepatic steatosis and fat droplets in the hepatocytes of HFD diet-fed mice, while FA treatment ameliorated these changes (Figures 1F,G). Consistent with the above phenomenon, FA supplementation markedly reduced the content of TG in the liver (Figure 1H). Simvastatin, which can improve lipid metabolic disorders, was used as a control to evaluate the effect of FA. The results showed that FA attenuated lipid metabolism in a manner consistent with simvastatin. Collectively, these data suggest that FA intervention significantly ameliorated lipid accumulation in the livers of HFD-induced mice.

### 3.2 FA reduces FFA-induced lipid accumulation in hepatocytes

**Figure 2:**
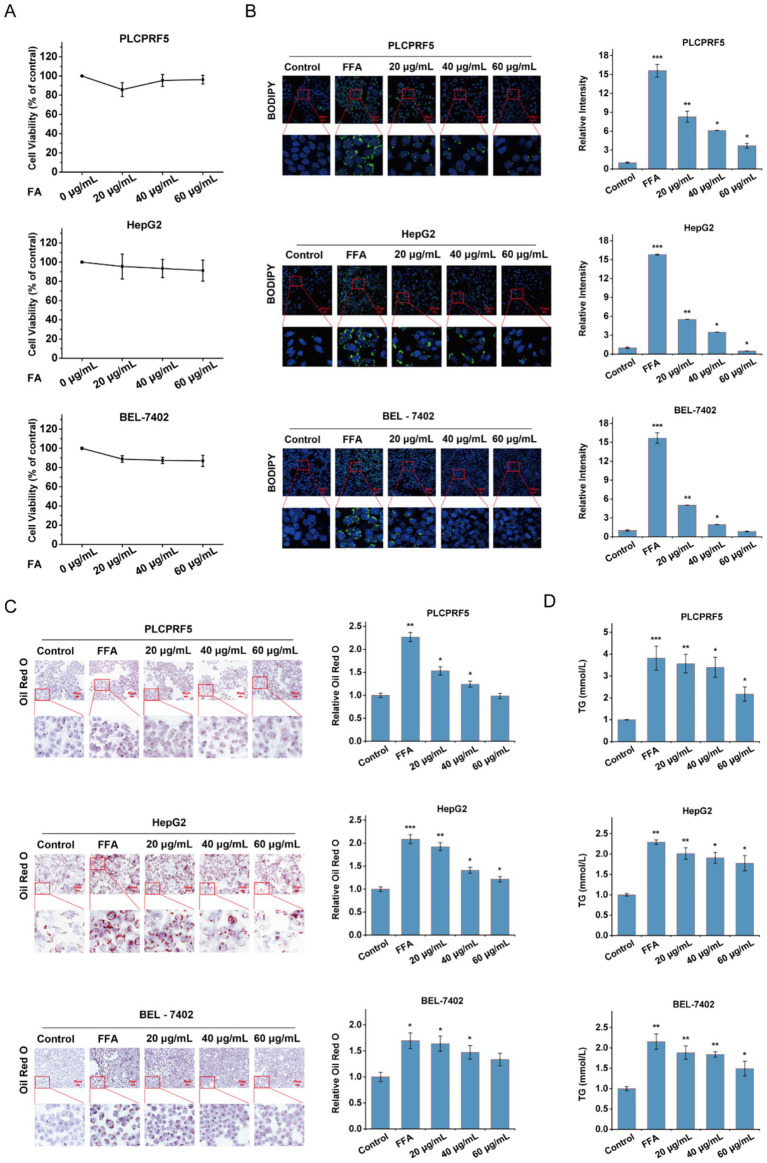
Effects of FA on lipid accumulation in FFA-induced hepatocytes. **(A)** The PLCPRF5, HepG2, and BEL-7402 cells were exposed to increasing concentrations of FA for 48 h; the cell viability was then detected using MTT. **(B,C)** After stimulation with or without FFA, PLCPRF5, HepG2, and BEL-7402 cells were exposed to FA and then stained with BODIPY **(B)** and Oil Red O **(C)** to detect cellular lipid accumulation. Representative images were shown, and quantitative analyses were performed. **(D)** TG contents in PLCPRF5, HepG2, and BEL-7402 cells of each group were treated with FA. The data are presented as the mean ± SEM from three independent experiments. *p < 0.05, **p < 0.01, ***p < 0.001.

We then examined the effects of FA in hepatocytes undergoing lipid accumulation. The results of MTT assays revealed that no cytotoxicity was observed at concentrations below 60 μg/mL (Figure 2A). BODIPY and Oil Red O staining further demonstrated that lipid accumulation in PLCPRF5, HepG2, and BEL-7402 cells was significantly decreased by FA intervention (Figures 2B,C). The decreased triglyceride levels also suggested that lipid accumulation in hepatocytes was alleviated following FA treatment (Figure 2D). These results emphasize that FA effectively alleviates lipid accumulation in FFA-treated hepatocytes.

### 3.3 PGC-1β is the target of FA intervention in fatty liver

**Figure 3:**
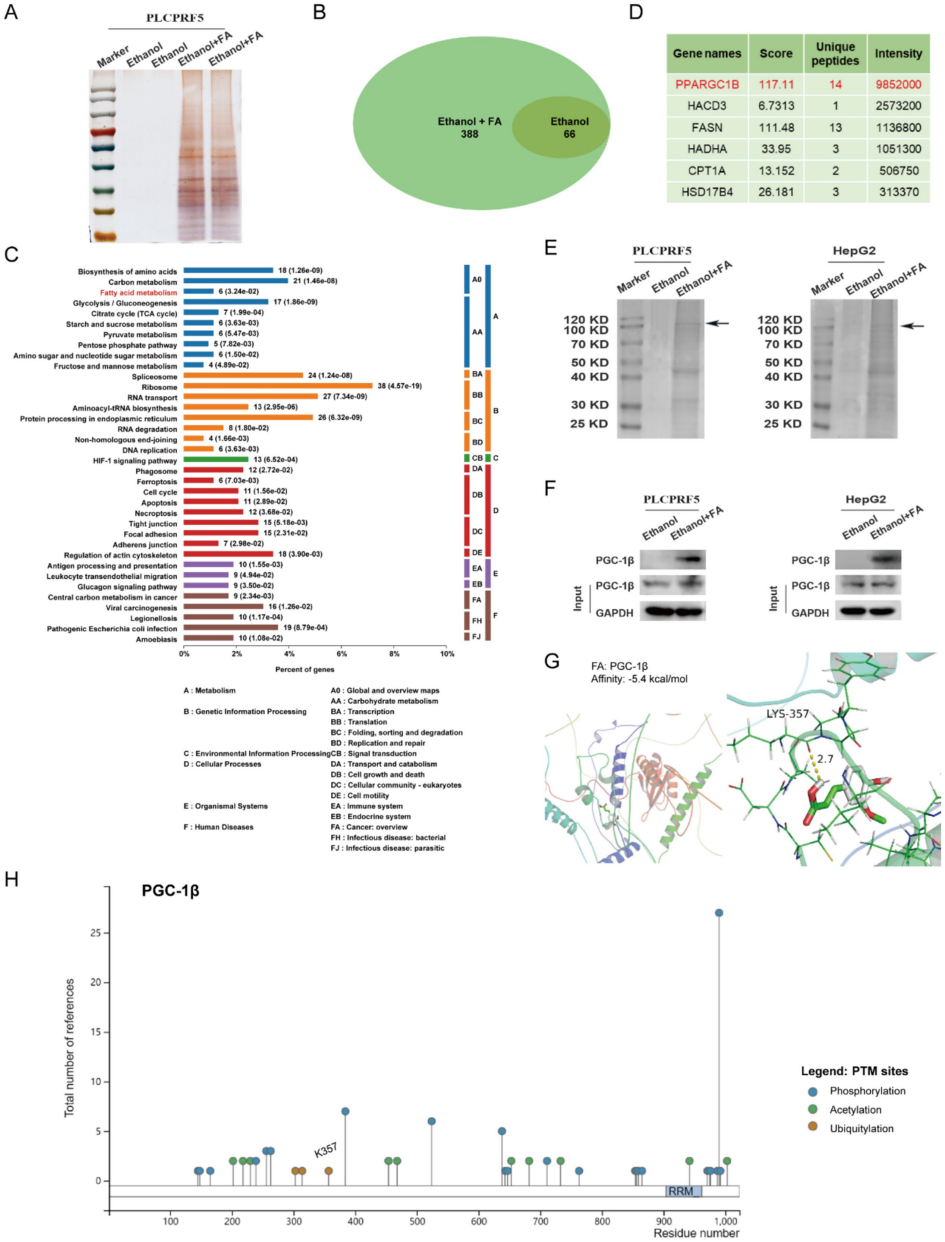
FA interacts with PGC-1β protein. **(A)** Proteins retrieved from the solid-phase extraction assay were analyzed using silver staining. **(B)** The Venn diagram shows the number of target proteins of FA. **(C)** The KEGG knowledge base was used to analyze the target proteins of FA. **(D)** The protein is enriched in the fatty acid metabolic pathway. **(E)** FA bound to PGC-1β protein is identified by solid-phase extraction and SDA-PAGE. **(F)** FA bound to PGC-1β protein is identified by solid-phase extraction and Western blotting assays. **(G)** Molecular docking model of FA and PGC-1β protein. **(H)** Post-translational modifications of PGC-1β proteins were predicted using PhosphoSitePlus.

To further investigate the molecular mechanisms underlying FA effects on FFA-induced lipid accumulation, the target proteins of FA in FFA-induced PLCPRF5 were identified using a solid-phase extraction assay. Silver staining assay revealed that the FA group had specific binding proteins compared to the ethanol group (Figure 3A). These proteins were analyzed by mass spectrometry, and 322 FA-specific binding proteins were identified (Figure 3B). KEGG enrichment analysis revealed that the target proteins of FA were enriched in metabolic pathways, including the fatty acid metabolic pathway closely related to MASLD (Figure 3C). PPAR gamma-coactivator-1beta (*PPARGC1β*, also known as PGC-1β), the most enriched in the above pathway, is considered to be the target protein of FA (Figure 3D). To substantiate the interaction between FA and PGC-1β protein, a solid-phase extraction assay was used. Sodium dodecyl sulfate–polyacrylamide gel electrophoresis (SDS-PAGE) analysis showed that a distinct protein band of 100 kDa, which is consistent with the size of PGC-1β protein, was observed in the FA group compared to the ethanol group (Figure 3E). The results of Western blotting further confirmed the binding of FA to PGC-1β (Figure 3F). Interestingly, molecular docking analysis showed that PGC-1β possessed binding pockets for FA binding, with a binding score of −5.4 kcal/mol, and that FA forms hydrogen bonds with the K357 site of PGC-1β (Figure 3G). Moreover, PhosphoSitePlus prediction identified that K357 is the ubiquitination modification site of PGC-1β (Figure 3H). These results indicate that PGC-1β is a direct target of FA.

### 3.4 FA decreases the stability of PGC-1β by the ubiquitin–proteasome pathway

**Figure 4:**
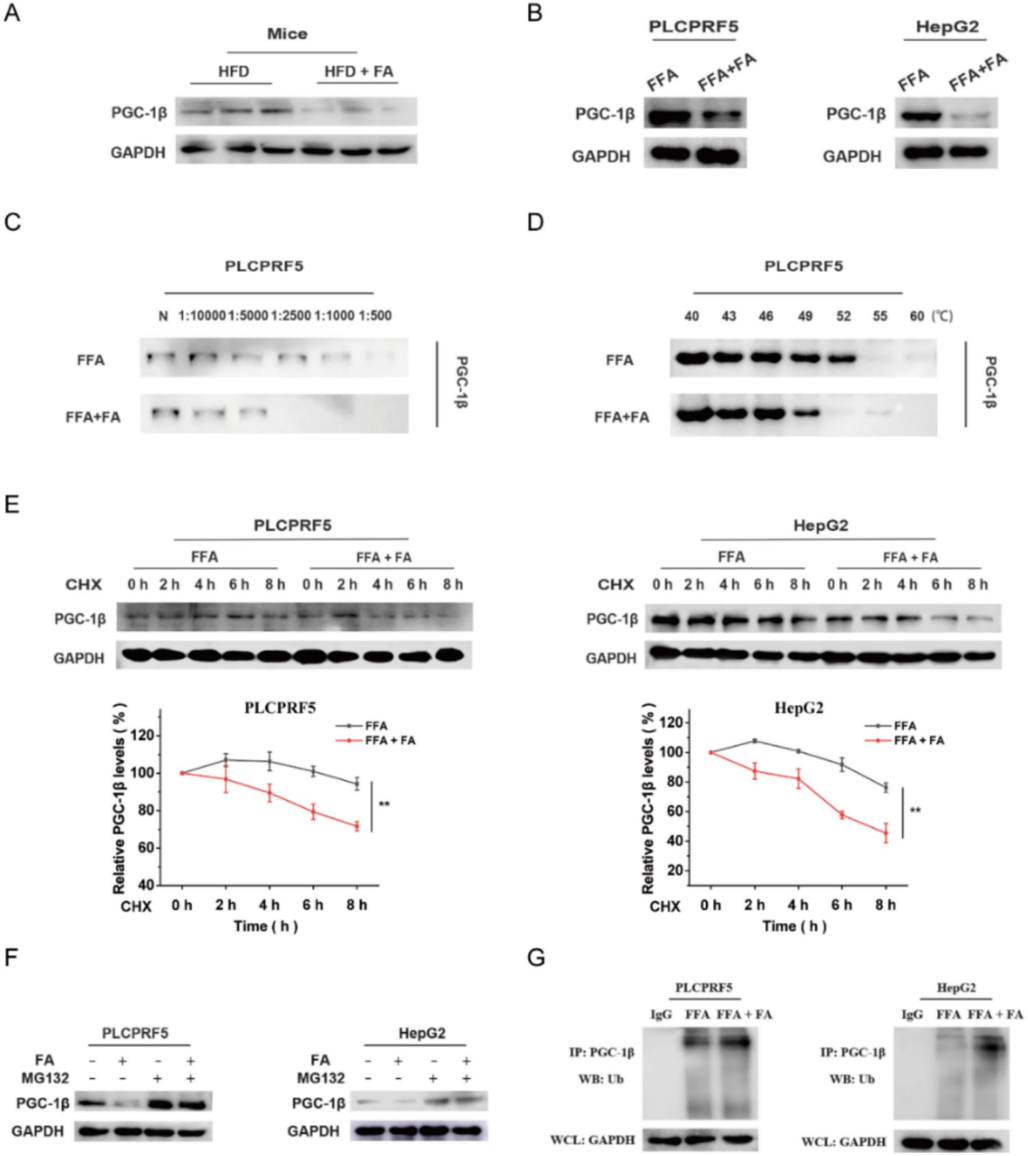
FA decreases PGC-1β protein levels by the ubiquitin–proteasome pathway. (A, B) The protein expression of PGC-1β after FA treatment was detected *in vivo* **(A)** and *in vitro* **(B)**. **(C,D)** DARTS **(C)** and CETSA **(D)** assays were used to detect the effect of FA on protein stability. **(E)** Cells were treated with CHX (20 μg/mL) for 2, 4, 6, and 8 h before harvest. The protein expression of PGC-1β was checked. The densitometry analysis of relative protein expression is shown at the bottom. **(F)** The protein expression of PGC-1β was measured in PLCPRF5 and HepG2 cells. The cells were incubated with MG132 (20 μM) for 6 h before harvest. **(G)** *In vitro* ubiquitination assay was performed in PLCPRF5 and HepG2 cells with or without FA. All cells were treated with MG132 (20 μmol/L) for 6 h. The data are presented as the mean ± SEM from three independent experiments. *p < 0.05, **p < 0.01.

Based on the above results, we hypothesized that FA could regulate the PGC-1β protein through the ubiquitin–proteasome pathway. The results indicated that PGC-1β protein expression was significantly decreased after FA intervention (Figures 4A,B). Moreover, DARTS and CETSA assays showed that FA reduced the stability of the PGC-1β protein (Figures 4C,D). Meanwhile, the protein synthesis inhibitor CHX was used to evaluate the effect of FA on the degradation of PGC-1β and showed that the half-life of PGC-1β was shortened after FA intervention (Figure 4E). Furthermore, the proteasome inhibitor MG132 prevented PGC-1β downregulation following FA intervention (Figure 4F), suggesting that FA regulates the expression of PGC-1β through the ubiquitin–proteasome pathway. To further validate these data, we evaluated the effect of FA on PGC-1β ubiquitination using an *in vitro* ubiquitination assay. The results revealed that FA reduced the stability of PGC-1β by increasing the ubiquitous modification of PGC-1β (Figure 4G). The results indicate that FA inhibited the expression of PGC-1β protein through the ubiquitin–proteasome pathway.

### 3.5 PGC-1β mediates the ameliorating effect of FA on MASLD

**Figure 5:**
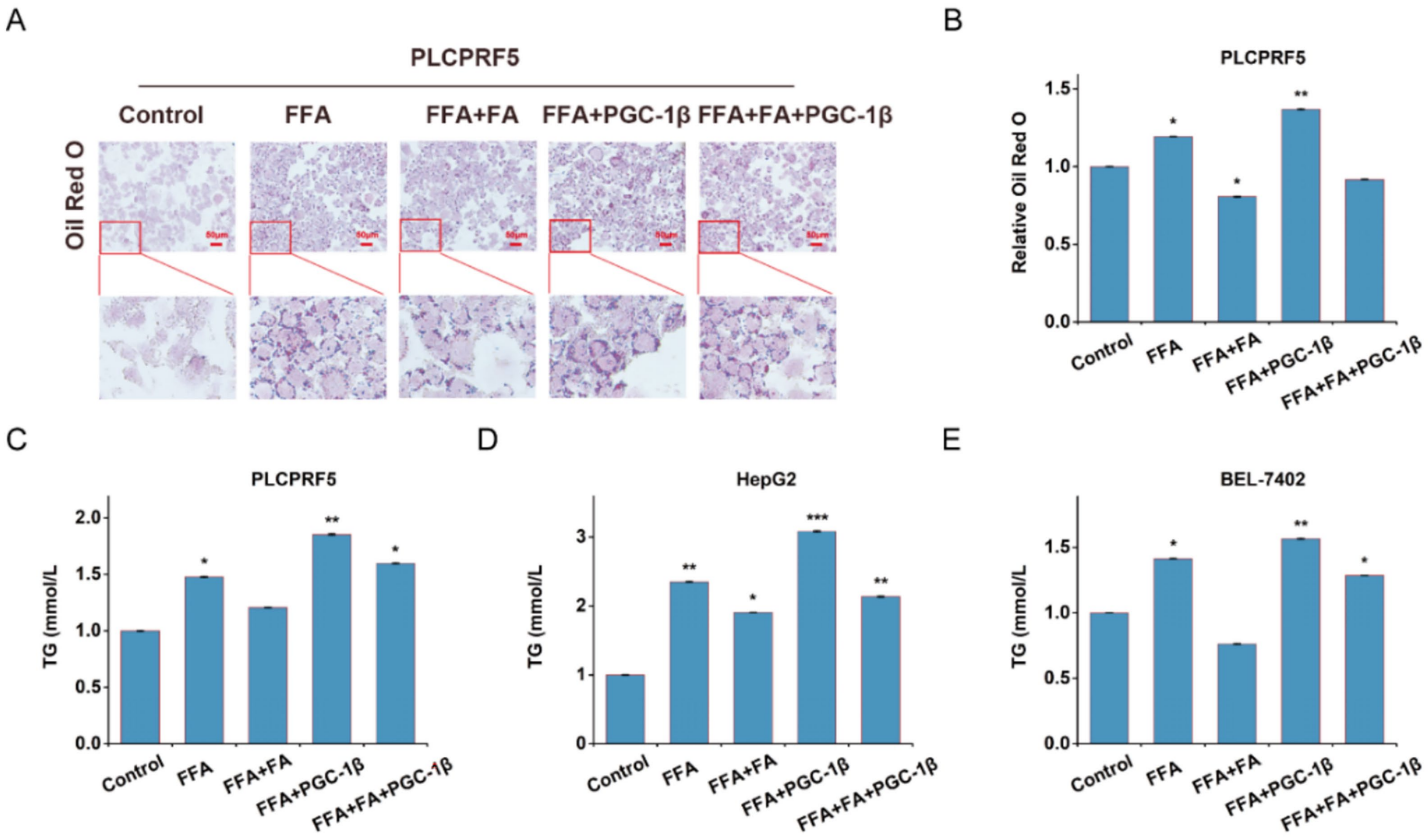
PGC-1β mediates the ameliorating effect of FA on MASLD. At 24 h post-transfection with GFP or PGC-1β, cells were treated with 40 μg/mL FA for another 48 h. **(A, B)** Representative images of Oil Red O staining were shown. Representative images were shown **(A)** and quantitative analyses were performed (B). (C-E) TG content in PLCPRF5 (C), HepG2 (D) and BEL-7402 (E) cells. The data were presented as the mean±SEM from three independent experiments. *p < 0.05, **p < 0.01.

To test whether PGC-1β is involved in the improvement effect of FA against fatty liver, we overexpressed PGC-1β protein before 40 μg/mL FA in FFA-induced hepatocytes. Oil Red O staining showed that overexpression of PGC-1β protein promoted FFA-induced lipid accumulation and blocked the improvement effect of FA on lipid accumulation (Figures 5A,B). Furthermore, FA treatment decreased TG content in FFA-induced hepatocytes, whereas the overexpression of PGC-1β protein partially counteracted the inhibitory effect of FA on TG content (Figures 5C–E). These results suggest that overexpression of PGC-1β protein eliminates the beneficial effect of FA treatment on fatty liver.

### 3.6 FA inhibits lipogenesis through the PGC-1β/SREBP1 axis

**Figure 6:**
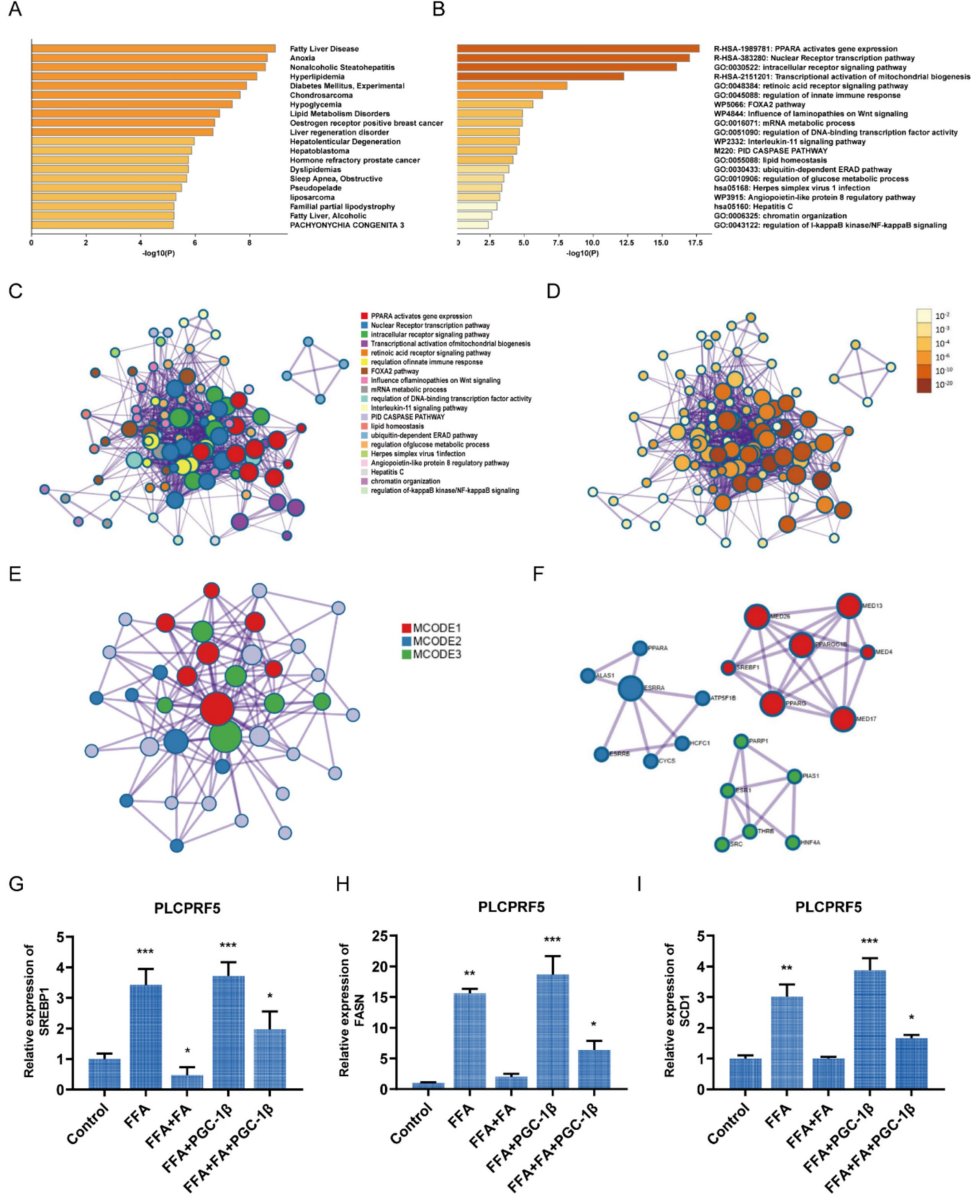
FA inhibits lipogenesis through the PGC-1β/SREBP1 axis. The proteins interacting with PGC-1β were imported into Metascape for gene enrichment analysis. **(A)** Summary of enrichment analysis in DisGeNET. **(B)** The top-level Gene Ontology biological processes. **(C)** Network of enriched terms, colored by cluster ID, where nodes that share the same cluster ID are typically close to each other. **(D)** Network of enriched terms, colored by *p*-value, where terms containing more genes tend to have a more significant p-value. (E, F) Protein–protein interaction network **(E)** and MCODE components **(F)**. **(G-I)** At 24 h post-transfection with GFP or PGC-1β, the cells were treated with 40 μg/mL FA for another 48 h. The expression of SREBP1 **(G)**, FASN **(H)**, and SCD1 **(I)** was measured. The data are represented as means ± SEM. *p < 0.05, **p < 0.01.

To investigate the molecular mechanism by which FA regulates non-alcoholic fatty liver through PGC-1β, proteins interacting with PGC-1β were predicted using the BioGRID Database and the IntAct Molecular Interaction Database. These target proteins were imported into Metascape for gene enrichment analysis. The results revealed that the proteins interacting with PGC-1β were enriched in pathways related to fatty liver disease (Figure 6A) and were involved in PPARA, thereby activating gene expression, the nuclear receptor transcription pathway, and the intracellular receptor signaling pathway (Figures 6B–D). The protein–protein interaction network and Molecular Complex Detection (MCODE) analysis indicated that PGC-1β targets proteins were involved in PPARA-activated gene expression, PPARA-mediated regulation of lipid metabolism, and transcriptional regulation of white adipocyte differentiation (Table 1, Figure 6E). PGC-1β is known to function as a transcriptional coactivator of SREBP-1, a key regulator of liver adipogenesis (7). As shown in Figure 6F, an interaction between PGC-1β and SREBP1 was observed. Furthermore, overexpression of PGC-1β protein promoted the expression of SREBP1 and blocked the inhibitory effect of FA on SREBP1 (Figure 6G). FA decreased the expression of FASN and SCD1 genes, the key enzymes of lipogenesis, while overexpression of PGC-1β protein partially counteracted the inhibitory effect of FA on FASN and SCD1 (Figures 6H,I). Based on this study, ferulic acid, through the PGC-1β/SREBP1 axis, inhibits lipogenesis to improve non-alcoholic fatty liver.

**Table 1:** MCODE components identified in the proteins interacting with PGC-1β.

MCODE	GO	Description	Log10(P)
MCODE_1	R-HSA-1989781	PPARA activates gene expression	−17
	R-HSA-400206	Regulation of lipid metabolism by PPARalpha	−16.9
	R-HSA-381340	Transcriptional regulation of white adipocyte differentiation	−14.6
MCODE_2	R-HSA-2151201	Transcriptional activation of mitochondrial biogenesis	−15.7
	R-HSA-1592230	Mitochondrial biogenesis	−14.2
	R-HSA-1852241	Organelle biogenesis and maintenance	−11.2
MCODE_3	GO:0030522	Intracellular receptor signaling pathway	−7.9
	GO:0006351	DNA-templated transcription	−7.8
	GO:0097659	Nucleic acid-templated transcription	−7.8
	GO:0097659	Nucleic acid-templated transcription	−7.8

## 4 Discussion

MASLD has become the most common chronic liver disease worldwide and a major public health concern. Drugs developed for MASLD treatment have shown serious adverse effects in clinical trials (20, 21), resulting in a lack of approved treatments. Therefore, much attention has been focused on the use of natural compounds to improve MASLD. Caffeic acid phenethyl ester derived from propolis improves MASLD and inhibits intestinal FXR signaling (22). Plant-derived sulforaphane improves MASLD by promoting the FGF21/FGFR1 signaling pathway (23). FA is an active plant-derived ingredient with a variety of biological activities (24). Among them, the intervention effect of FA on lipid metabolism gained our attention. In addition, FA has been approved by the National Medical Products Administration (NMPA) for the treatment of cardiovascular and cerebrovascular diseases (25). In this study, the improvement effect of FA on MASLD was evaluated using an HFD-induced MASLD model, which mimics the histological features of MASLD in humans. FA significantly improved HFD-fed mice exhibiting typical features of MASLD, such as dyslipidemia, lipid accumulation, and hepatocyte ballooning (Figure 1). *In vitro* assays further indicate that FA improves lipid accumulation in hepatocytes without significant toxic side effects (Figure 2). These results suggest that FA may serve as a drug candidate for the treatment of MASLD.

The majority of drugs work by interacting with specific biological target molecules, and their therapeutic effect largely depends on these targets. For example, Xiao et al. reported that gentiopicrin targets PAQR3, a key regulator of inflammation and metabolism, to improve glucose and lipid metabolism disorders (26). IMA-1 targets ALOX12 to inhibit lipid uptake and ameliorate non-alcoholic steatohepatitis (27). Nuciferine targets HBXIP to inhibit mTORC1 and activate the TFEB-mediated autophagy-lysosome pathway, thereby further improving hepatic steatosis (28). In this study, we found that FA target proteins were enriched in the fatty acid metabolism pathway. We selected PGC-1β protein, which showed the highest enrichment in fatty acid metabolism, as a target protein for FA intervention in MASLD (Figure 3). Further studies revealed that FA interacts with PGC-1β and inhibits its expression through the ubiquitin–proteasome pathway (Figure 4). Interestingly, overexpression of PGC-1β diminished the beneficial effect of FA on lipid accumulation (Figure 5). Notably, we identified K357 as the key residue mediating the FA-PGC-1β interaction. However, we acknowledge a limitation of our study: the identification of K357 was based on bioinformatic prediction and molecular docking. While these computational approaches provide strong supportive evidence, definitive functional validation through site-directed mutagenesis of this residue will be essential in future studies to conclusively establish its role. Despite this, the collective evidence strongly supports the conclusion that PGC-1β serves as a direct and functional target for FA in improving MASLD, highlighting its potential as a promising candidate for further therapeutic exploration.

PGC-1β belongs to the PGC-1 coactivator family. It is a transcriptional coactivator that stimulates the activity of various transcription factors and nuclear receptors and participates in multiple metabolic pathways. PGC-1β plays a key role in promoting adipogenesis (29–31). In our study, we screened proteins that interact with PGC-1β. The results displayed that there was an interaction between PGC-1β and SREBP1 (Figure 6), which is a major regulator of liver adipogenesis. It has been reported that PGC-1β synergistically activates SREBPs and significantly promotes the expression of SREBP-dependent lipogenic genes in the liver (32). The results shown in Figures 6H,I indicate that PGC-1β increases the expression of FASN and SCD1, key enzymes of lipogenesis, whereas ferulic acid treatment inhibited their expression. These findings confirm that ferulic acid inhibits lipogenesis through the PGC-1β/SREBP1 axis, thereby improving non-alcoholic fatty liver.

## 5 Conclusion

FA significantly ameliorated hepatic steatosis in both the HFD-induced MASLD mouse model and FFA-treated hepatocytes. Further study showed that FA target genes were enriched in fatty acid metabolism pathways, with PGC-1β being the most enriched protein. Mechanistically, PGC-1β serves as the target of FA, which directly binds to and inhibits its expression via the ubiquitin–proteasome pathway (Graphical abstract). Moreover, FA inhibits lipogenesis through the PGC-1β/SREBP1 axis, thereby improving MASLD. These findings demonstrate that FA suppresses hepatic lipid accumulation by targeting PGC-1β, suggesting that it could be a promising agent for the treatment or remission of MASLD.

## Data Availability

The datasets presented in this study can be found in online repositories. The names of the repository/repositories and accession number(s) can be found in the article/Supplementary material.
