# Supplementary material for: Ferulic acid inhibits lipogenesis and ameliorates MASLD via targeting PGC-1β
Source: Front Nutr. 2026 Jan 5;12:1730916. doi: 10.3389/fnut.2025.1730916 (PMC12813010; doi:10.3389/fnut.2025.1730916)
Supplement: Supplementary file 1 [file Image_1.pdf]

Supporting Information

Supplement Figure 1

Ferulic acid

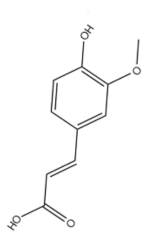

Supplement Figure 1. The chemical structure of Ferulic acid.
