# Supplementary material for: Ferulic acid inhibits lipogenesis and ameliorates MASLD via targeting PGC-1β
Source: Front Nutr. 2026 Jan 5;12:1730916. doi: 10.3389/fnut.2025.1730916 (PMC12813010; doi:10.3389/fnut.2025.1730916)
Supplement: Supplementary file 2 [file Image_2.pdf]

Supporting Information

Supplement Figure 2

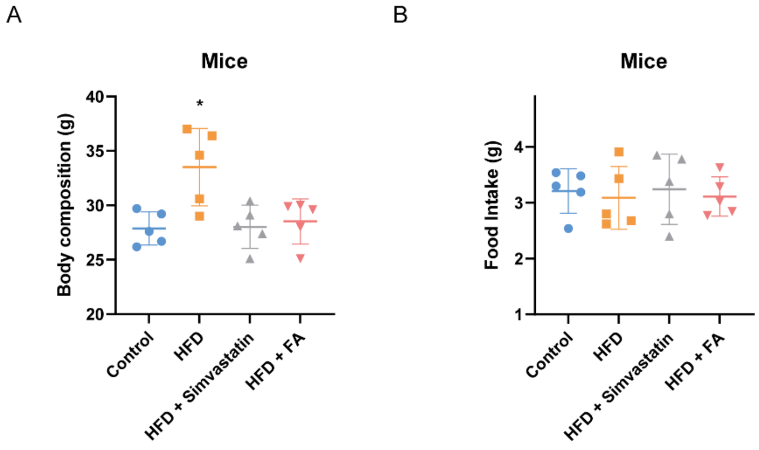

Supplement Figure 2. Effects of FA on HFD-induced hepatic steatosis. (A) Body composition of all experimental groups (n = 5). (B) Food intake.
