# Supplementary material for: Ferulic acid inhibits lipogenesis and ameliorates MASLD via targeting PGC-1β
Source: Front Nutr. 2026 Jan 5;12:1730916. doi: 10.3389/fnut.2025.1730916 (PMC12813010; doi:10.3389/fnut.2025.1730916)
Supplement: Supplementary file 3 [file Table_1.pdf]

Supplement Table 1. Primers sequences used for qRT-PCR.

Genes	Primer Sequences (5′−3′)
**SREBP1 R**	AAGGAGACGAGCACCAACAG
**SREBP1 F**	CAGCGTCTACCATAGCCCTG
**FASN R**	CACATTTCAAAGGCCACGCA
**FASN F**	CCTGGCTGCCTACTACATCG
**SCD1R**	CCGGGGGCTAATGTTCTTGT
**SCD1 F**	CTTGCGATATGCTGTGGTGC
**GAPDH R**	TGGTGAAGAACGCCAGTGGA
**GAPDH F**	GCACCGTCAAGGCTGAGAAC
